# THE OBESITY BILL OF RIGHTS: A FRAMEWORK FOR HEALTH EQUITY AND SELF-ADVOCACY

**DOI:** 10.1093/geroni/igae098.1702

**Published:** 2024-12-31

**Authors:** Gretchen Tanbonliong

**Affiliations:** Chair

## Abstract

When combined with common aging-related challenges, obesity, which is now recognized as a chronic condition, can create complex clinical situations. A particularly challenging factor is the issue of stigma from weight bias and ageism, both of which are significant barriers to meeting the individual needs of the person with obesity/overweight. The Obesity Bill of Rights (OBOR) was created in response to the urgent need to pave the way for a more equitable landscape where every person is respected and guaranteed unbiased, comprehensive management and treatment of obesity. This symposium will present multiple stakeholder viewpoints that led to the research and development of the OBOR; how it is operationalized in health care delivery settings; and how providers and patients may work collaboratively to achieve the research-informed principles articulated in the OBOR.

